# Dirty deeds and dirty bodies: Embodiment of the Macbeth effect is mapped topographically onto the somatosensory cortex

**DOI:** 10.1038/srep18051

**Published:** 2015-12-21

**Authors:** Michael Schaefer, Michael Rotte, Hans-Jochen Heinze, Claudia Denke

**Affiliations:** 1 Department of Neurology, Otto-von-Guericke University Magdeburg, 39120 Magdeburg, Germany; 2 Department of Anesthesiology and Intensive Care Medicine, Charité – Universitätsmedizin Berlin, Berlin, Germany

## Abstract

The theory of embodied cognition claims that knowledge is represented in modal systems derived from perception. Recent behavioral studies found evidence for this hypothesis, for example, by linking moral purity with physical cleansing (the Macbeth effect). Neurophysiological approaches provided further support by showing an involvement of sensorimotor cortices for embodied metaphors. However, the exact role of this brain region for embodied cognitions remains to be cleared. Here we demonstrate that the involvement of the sensorimotor cortex for the embodied metaphor of moral-purity is somatotopically organized. Participants enacted in scenarios where they had to perform immoral or moral acts either with their mouths or their hands. Results showed that mouthwash products were particularly desirable after lying in a voice mail and hand wash products were particularly desirable after writing a lie, thus demonstrating that the moral-purity metaphor is specific to the sensorimotor modality involved in earlier immoral behavior. FMRI results of this interaction showed activation in sensorimotor cortices during the evaluation phase that was somatotopically organized with respect to preceding lying in a voice mail (mouth-area) or in a written note (hand-area). Thus, the results provide evidence for a central role of the sensorimotor cortices for embodied metaphors.

When thinking about religious ceremonies a close relationship between moral transgressions and physical cleanliness is well known. For example, baptism is an essential part of Roman Catholic, Protestantism and other churches. Or think about the rule to clean yourself before entering a mosque. Another example is the use of holy water to bless oneself in Roman Catholic churches. In the last years psychological experiments investigated this link systematically. Experiments by Zhong and Liljenquist^1^ revealed that hand washing removes the guilt of past misdeeds. For example, one of their experiments showed that copying an unethical story increased the desirability of cleansing products as compared to copying an ethical story^1^,^2^. In addition, Schnall *et al.*^3^ reported that cleaning products soften one’s judgment of others’ misdeeds. Furthermore, hand washing also seems to reduce postdecisional dissonance^4^.

So why does hand washing remove more than dirt? The results may be explained by the conceptual metaphor theory, which suggests that many constructs in social cognition are metaphorical^5^. According to this theory of embodiment cognitive representations are structured by metaphorical mappings from sensory experience. Thus, abstract thoughts about morality may be grounded in concrete experiences of physical cleanliness^2^,^5^. Numerous studies provide support for the theory of embodied cognition. For example, Williams and Bargh^6^ demonstrated that experiencing physical warmth, e.g., holding a cup of hot (versus iced) coffee, make it likely to judge a person as having a “warm” personality. Ackerman *et al.*^7^ showed that basic tactile sensations have an impact on higher social cognitive processing in dimension- and metaphor-specific ways. Hence, holding a heavy clipboard made job candidates appear more important^7^,^8^.

Only very recently studies using neuroimaging approaches tried to unravel the neural underpinnings of embodied metaphors. Kang *et al.*^9^ employed fMRI to examine effects of physical temperature on trust behavior and found activation in bilateral anterior insula, central operculum, and primary somatosensory cortex (SI) associated with the embodied metaphor of warmth. Desai *et al.*^10^ compared neural responses to literal and metaphoric action and found activity in left anterior inferior parietal lobe, suggesting that the understanding of a metaphoric action retains a link to sensory-motor systems engaged in action performance. Lacey *et al.*^11^ examined textural metaphors and reported activation of texture-selective-somatosensory cortex in the parietal operculum. Saxbe *et al.*^12^ examined language use during the feeling of social emotions and found somatosensory activity linked with complex social emotional processing. Our previous study examined the metaphor roughness and showed that rough tactile priming made social interactions appear more difficult and adversarial, consistent with the rough metaphor. This effect was accompanied with a cortical network including in particular the somatosensory cortices^13^, (see also^14^).

In accordance with the assumptions of the theory of embodied cognition the above-mentioned studies found an involvement of sensorimotor brain areas when examining the neural correlates of embodied metaphors. However, the exact role of the sensorimotor cortices for embodied cognitions remains to be cleared.

A recent experiment by Lee and Schwarz^2^ offers the opportunity to characterize the role of the sensorimotor cortices for embodied metaphors more in detail. Lee and Schwarz^2^ point out that natural language use associates the moral-purity metaphor with specific body parts, e.g., “dirty hands” or “dirty mouth”. In a behavioral experiment the authors showed that people prefer purification of the “dirty” body part of other body parts. They conclude that the embodiment of the moral-purity metaphor is specific to the motor modality involved in moral transgression. The present study made use of this effect in order to determine the role of the sensorimotor cortices for embodied cognitions. Given that there is a well known functional topography in SI and primary motor cortex (M1) (also described as the somatosensory homunculus), we hypothesized that the preference for purification of the “dirty” body part is reflected in a somatotopical activation of the sensorimotor cortex. If the moral-purity metaphor is mapped topographically on SI (and M1), this result would provide strong support for the theory of embodiment and for a fundamental role of the sensorimotor cortices for embodied or grounded cognitions.

In order to test our hypothesis if embodiment is sensitive to motor modality we adopted the paradigm of Lee and Schwarz^2^ for an fMRI experiment. While scanning brain activity participants had to enact scenarios and behave either ethical (telling the truth) or not ethical (lying). This was done either by hand (writing) or by mouth (speaking). Subsequent assessments of the desirability should evoke increased ratings for hand wash or toothpaste and mouthwash products, depending on the previous modality of moral transgressions. We hypothesized that these preferences in purification were accompanied by an engagement of the hand- or mouth area in sensorimotor cortex, respectively.

## Materials and Methods

### Participants

35 people (17 females) with a mean age of 25 years (± 3.77, range 20–35 years) took part in the study. All participants were right-handed native German volunteers with no neurological or psychiatric history. The participants gave written informed consent to the study, which adhered to the Declaration of Helsinki and was approved by the local human subjects’ committee.

### Procedure

Participants were told that they would perform two separate experiments in the fMRI scanner. The first one was described as a cognitive neuroscience experiment. The second one was introduced as not linked to the previous one and having a marketing background. All participants were naive to the real aim of the study. At the end of the experiment participants were probed for suspicions about the experiment’s true purpose.

The study consisted out of a three-factorial experimental design. The first factor was ethicality (ethical or unethical behavior). The second factor described the modality (writing a note with the hand (hand) or leaving a voicemail (mouth)). The third factor was the set of products participants had to rate with respect to the desirability (toothpaste and mouthwash products vs. hand soaps).

While lying in the fMRI participants enacted short scenarios modeled analogue to Zhong and Liljenquist^1^ and Lee and Schwarz^2^. For example, participants read the following scenario: “Imagine you are a law-firm associate competing for promotion with a colleague Sven. Today in the morning you found an important document on the floor by accident, which has obviously been lost by your colleague Sven. Returning this document to Chris would be very important for Sven, but might hurt your own career.” After 18 seconds a new screen showed up, instructing the participant to leave Sven a voice mail (mouth condition) or a note with a pencil on a paper (hand condition). In the unethical voice mail condition (mouth condition) the participant was prompted with the following screen: “Please leave a voice mail for Sven. Tell him who you are and that you did not find his document. Please speak now!”. In the ethical voicemail condition (telling the truth) the participant was prompted with this instruction: “Please leave a voice mail for Sven. Tell him who you are and that you did find the document. Please speak now!” In the hand condition the participants were faced with the following instructions (ethical): “Please leave a note for Sven. Write him that you did not find his document and sign the note. Start now!”. For the ethical condition the instruction was like this: “Please leave a note for Sven. Write him that you did find his document and sign the note. Start now!”.

The participants had 17 seconds to perform this task. Then a new screen showed up asking the participants to rate the following products on a four-point scale (Likert-scale, 1 = completely undesirable, 4 = completely desirable). After 2.5 seconds two pictures of products were shown, each lasting for 4 seconds with an interstimulus interval of 9 seconds. Thus, the participants had 13 seconds to rate each of the products (earlier responding did not automatically start the next trial). One of the pictures was taken out of a category of hand soap products, the other picture belonged to a category of toothpastes and mouthwash products.

Participants were required to press buttons with their right hand using a key with four buttons (ranging from +2 to −2) to assess the products. Before the experiment they were explained that they could weight their responses from moderate (inner buttons) to extreme (outer buttons). Participants were made familiar with both tasks before beginning of the experiment.

The experiment consisted out of a total of 60 scenarios. Each scenario was repeated two times, followed by either the instruction to convey a malevolent or the instruction to give a benevolent message. Half of the scenarios included the instruction to leave a written message, the other half asked the participants to leave a message on the voice mail. The order of presentation of the two product categories (first or second place) was randomized. Furthermore, the products for each category were randomized over all conditions (within and between subjects). Products were chosen as equally attractive in a pre-study. Participants were told that the voice messages inside the scanner were recorded for later analysis. In fact we did not record these messages.

Visual images were back-projected to a screen at the end of the scanner bed close to the subject’s feet. Subjects viewed the images through a mirror mounted on the birdcage of the receiving coil. Foam cushions were placed tightly around the side of the subject’s head to minimize head motion.

The experiment consisted out of six runs, each lasting for about 12 minutes and including all conditions. Participants were allowed to take short breaks between the runs. All data were tested for normality of distribution and possible outliers^15^.

### FMRI Data Acquisition and Analysis

Data acquisition was done on a 3 T scanner (Siemens MAGNETOM Trio, Germany). T2-weighted functional images were acquired using gradient echo-planar images (TR = 2 sec, TE = 35 ms, flip angle = 80 degrees, FOV = 224 mm). For each subject, data were acquired in six runs. In each session, 404 volumes were acquired. Functional volumes consisted of 32 slices. Each volume comprised 3.5 mm slices (no gap, in plane voxel size 3.5 × 3.5 mm). For anatomic reference a high-resolution T1-weighted structural image was acquired using an MP-RAGE sequence (TR = 1650 ms, TE = 5 ms).

Image preprocessing and analysis was performed using the Statistical Parametric Mapping Software (SPM8, Wellcome Department of Imaging Neuroscience, University College London, London, UK). Individual functional images were realigned to correct for inter-scan movement using sinc interpolation and subsequently normalized into a standard anatomical space (MNI, Montreal Neurological Institute template) resulting in isotropic 3 mm voxels. Data were smoothed with a Gaussian kernel of 6 mm full-width half maximum.

We then computed statistical parametric maps by using multiple regressions with the hemodynamic response function modeled in SPM8. Data analyses were performed at two levels. First, we examined data on the individual subject level by using a fixed effects model. Second, the resulting parameter estimates for each regressor at each voxel were entered into a second-level analysis with the random effects model. In order to examine responses in sensorimotor brain areas when participants perform the (un) ethical task we calculated statistical contrasts (t-tests) for the time window while speaking relative to rest and writing relative to rest, respectively. In order to examine brain responses while assessing the products we examined the time window while evaluating the desirability of the products. We computed an ANOVA for repeated measurements with the factors previous ethical behavior (moral vs. immoral), set of products (toothpaste and mouthwash vs. hand soaps), and modality (hand vs. mouth). Statistical contrasts (t tests) were then performed to examine cortical activation associated with moral and immoral behavior for the different set of products and different modalities. In order to test our hypothesis that sensorimotor brain regions subserve the embodiment of the moral-purity metaphor in a topographic way, we masked these effects with results of the speaking and writing task, respectively.

Parameter estimates for voxels in the sensorimotor regions of interest (maximum peaks in SI and M1) were tested for possible correlations (Pearson) with behavioral responses (bias in rating of products). In addition to those brain areas we also tested possible correlations with BA6, hippocampus, and amygdala, since previous studies found additional activation in these regions (e.g.,^13^).

We report activations that survived a threshold of p < 0.05, family-wise error (FWE) corrected for multiple comparisons. Correction was achieved by imposing a threshold for the volume of clusters comprising contiguous voxels that passed a voxel-wised threshold of p < 0.005. Anatomical interpretation of the functional imaging results was performed by using the SPM anatomy toolbox^16^.

## Results

### Behavioral results

None of the participants reported any suspicions with respect to our experimental hypotheses when being asked at the end of the experiment.

**Figure 1:**
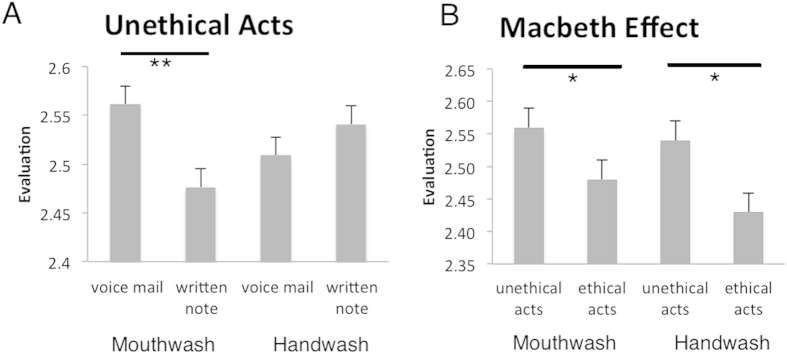
(**A**) Participants’ mean evaluation (+standard errors) of cleansing products after unethical (lying) acts (leaving a lie on a voicemail or lying in a written note). After lying in a voicemail toothpaste and mouthwash products were evaluated more positively than after lying in a written note. Analogue comparisons for evaluations after ethical behavior revealed no significant differences (all p > 0.10). (**B**) Participants’ mean evaluation of cleansing products after unethical (lying) or ethical acts (telling the truth). Cleansing products (toothpaste and mouthwash products as well as hand wash products) were evaluated significantly higher when participants performed an unethical deed before (the Macbeth effect).

An ANOVA with factors set of products, modality and ethics revealed a significant three-way interaction (F (1,34) = 4.82, p = 0.03, no other significant effects). Post hoc t-tests demonstrated that participants evaluated toothpaste and mouthwash products more positively after lying in a voicemail than after lying in a written note (voicemail: mean 2.56, standard deviation ± 0.53; written note: 2.48 ± 0.50; t(34) = 2.63, p = 0.006). Thus, toothpaste and mouthwash products were evaluated more positively only after lying in a voicemail (not after lying in a written note). Lying in a written note resulted in higher assessments for hand soaps compared with lying in a voicemail, but this difference failed to reach the level of significance (written note: 2.54, ± 0.43; voice mail: 2.51 ± 0.48; t(34) = 0.15, p = 0.26) (see Fig. 1). Analogue comparisons for evaluations after ethical behavior revealed no significant differences (all p > 0.10).

Furthermore, post hoc t-tests successfully replicated the Macbeth effect for both modalities (see Fig. 1). Thus, toothpaste and mouthwash products were evaluated more positively after lying compared with telling the truth in a voicemail (t(34) = 2.44, p = 0.01). In an analogue way, hand soap products were assessed more positively after lying relative to telling the truth in a written message (t(34) = 2.03, p = 0.02).

Analysis of the reaction times revealed no significant results.

### FMRI results: Brain responses while leaving a lie on a voicemail and as a written note

**Figure 2:**
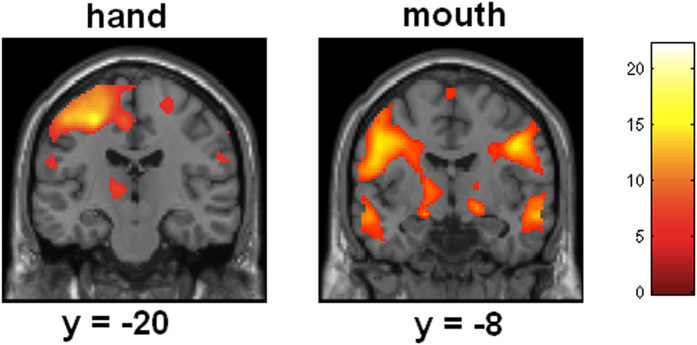
Statistical maps showing brain activation while participants were leaving a lie on a voicemail and while writing a lie on a note (relative to rest). Areas of significant fMRI signal change are shown as color overlays on the T1-MNI reference brain (FWE corrected at p > 0.05).

Brain responses when leaving a lie on a written note relative to rest revealed activations in left sensorimotor cortex, premotor brain areas, inferior frontal gyrus (BA44), right postcentral gyrus, middle temporal gyrus, right insula, inferior parietal cortex, superior parietal lobe, and occipital cortex. Leaving a lie as a voicemail was accompanied with activations in sensorimotor cortices, premotor cortex (BA44), superior parietal lobe (precuneus), and occipital cortex (at p < 0.05, FWE corrected). Comparing brain responses for writing relative to leaving a voicemail revealed more dorsal activation of sensorimotor brain regions than the contrast leaving a voicemail relative to writing, as expected (in line with the topography of the homunculus, see Fig. 2).

### FMRI results: Brain responses while evaluating products after lying in a voicemail

**Figure 3:**
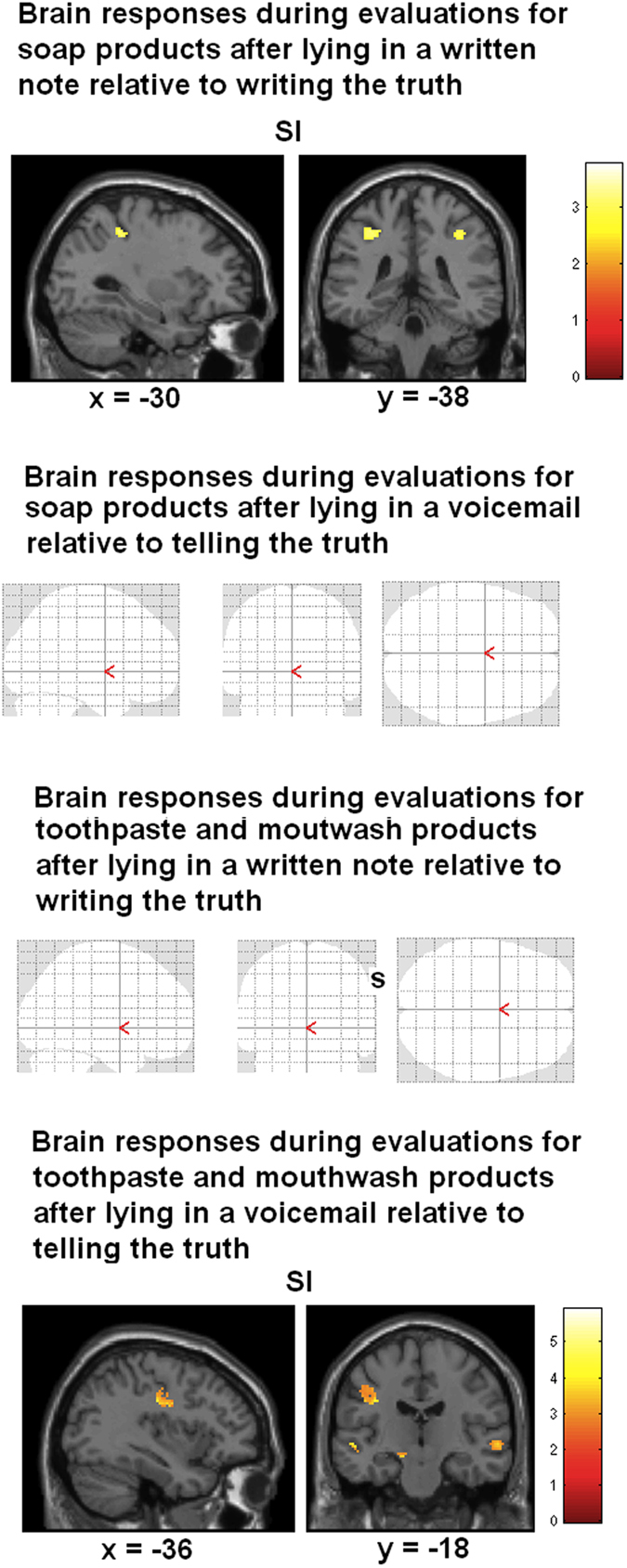
Statistical maps showing brain activation while participants had to evaluate hand soaps and toothpaste and mouthwash products. See text for details. Areas of significant fMRI signal change are shown as color overlays on the T1-MNI reference brain (FWE corrected at p > 0.05).

Results of the ANOVA (factors ethics, set of products and modality) revealed an interaction between the three factors, including activation of a cluster involving sensorimotor and premotor cortex (BA6), precuneus, anterior cingulate cortex, hippocampus, and occipital cortex. We then calculated brain responses after lying in a voicemail while evaluating different products (post-hoc t-tests). Comparing brain activations after lying relative to telling the truth (the Macbeth effect) for *toothpaste and mouthwash products* revealed activity in left sensorimotor and premotor cortex (masked with the contrast mouth-rest). The unmasked contrast revealed additional activation in right prefrontal gyrus and precuneus (MNI coordinates: 24 58 4, z = 4.25; −8 −82 50, z = 4.39; FWE corrected). As a further control condition we calculated brain responses for the same contrast, but masked with the contrast *hand-rest*. As expected, results revealed no significant voxels. The reverse contrast for telling the truth relative to lying revealed no significant activation (masked and unmasked). Furthermore, the analogue contrast lying relative to telling the truth (the Macbeth effect) for assessing *hand wash products* failed to reveal activity in sensorimotor cortex (masked with contrast mouth-rest). Unmasked results revealed activity in anterior cingulate cortex (−10 30 20, z = 6.35), bilateral insula (−30 14 −16, z = 5.75; 40 8 −6, z = 5.48), and inferior parietal lobe (58 −36 46, z = 4.98), but no activation of sensorimotor brain areas (see Table 1 and Fig. 3).

**Figure 4:**
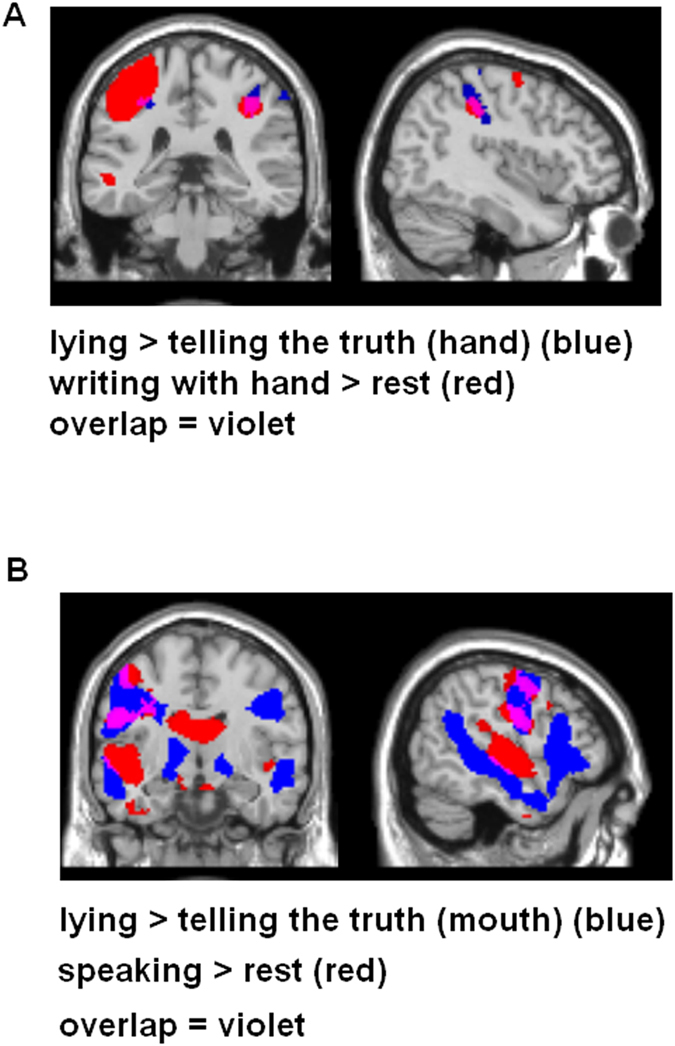
(**A**) Overlap (in violet) of brain activation while actually writing a lie (relative to rest, in red) with brain areas involved when participants had to evaluate hand soap products after writing a lie on a note (relative to telling the truth, in blue). (**B**) Same for the mouth modality (speaking a lie and assessment of toothpaste and mouthwash products).

Thus, previous lying in a voicemail resulted in sensorimotor engagement when subsequently evaluating mouthwash and toothpaste products, but not when rating hand wash products. This sensorimotor engagement was similar to the activity while performing the lying (mouth region of topography in sensorimotor cortex) (see Fig. 4).

**Figure 5:**
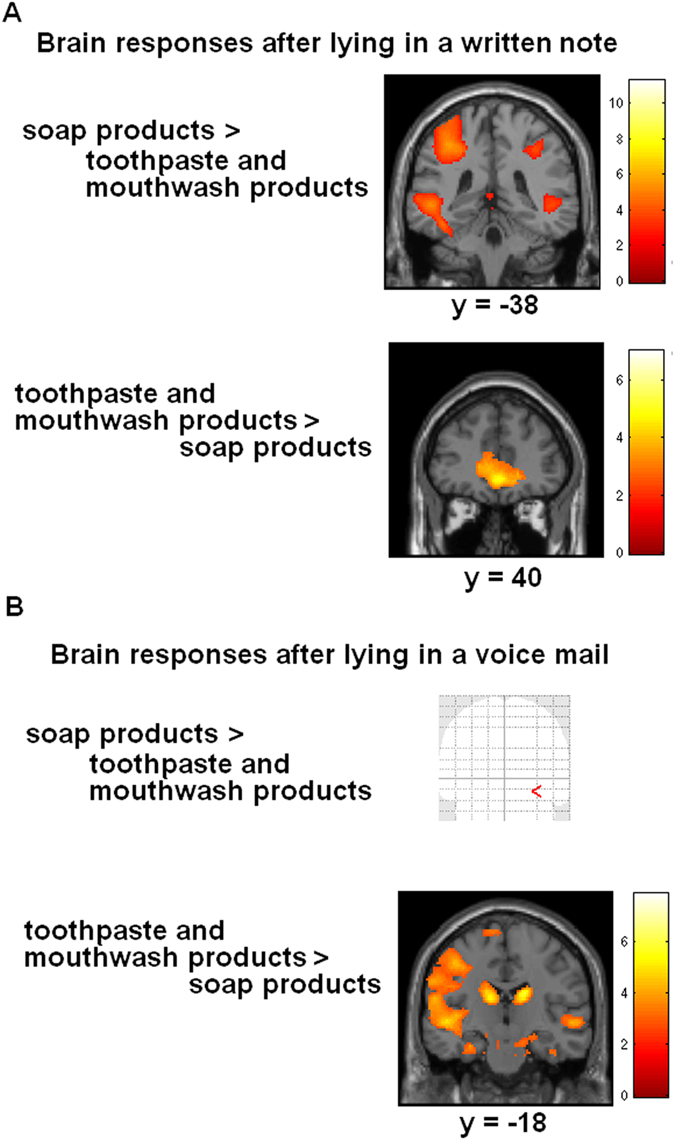
Statistical maps showing brain activation while participants assessed different sets of products after lying in a written note and after lying in a voicemail, respectively. Results demonstrate activations in sensorimotor brain areas after lying in a written note only while evaluating hand soaps, not mouthwash products. In addition, results show brain activation in sensorimotor cortices after lying in a voice mail only during assessment of mouthwash products, not hand soaps. Furthermore, the involvement of the sensorimotor cortices is different with respect to the hand and the mouth area of the functional topography in SI.

We then compared the effect of lying in a voice mail on the factor set of products. Results revealed involvement of left sensorimotor cortex when participants were evaluating toothpaste and mouthwash products, but no significant activation when they were rating soap products (unmasked results, FWE corrected, see Table 2 and Fig. 5). We concluded that the Macbeth effect *after speaking* a lie worked for toothpaste and mouthwash products, but not for hand soap products.

**Figure 6:**
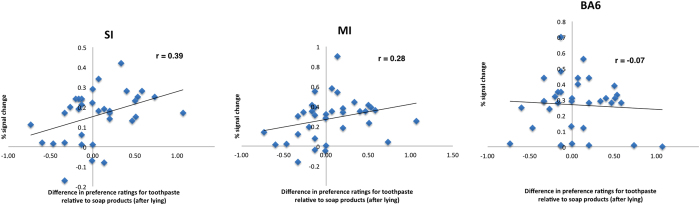
Scatterplots of BOLD responses in different brain regions while assessment of toothpaste and mouthwash relative to soap products after lying in a voicemail. Activity in SI could significantly predict the increased desirability of toothpaste and mouthwash products after unethical deeds (r = 0.39). Correlation of activity in M1 and BA6 (premotor cortex) failed to reach the level of significance.

Figure 6 displays a correlation between brain activations with signal changes in sensorimotor peak areas (SI and M1) associated with ratings for toothpaste and mouthwash products relative to hand wash products after lying in a voicemail and behavioral responses. Results demonstrate a significant positive correlation of r = 0.39 for SI (p = 0.02, Pearson, two-sided). The correlation for M1 failed to reach the level of significance (r = 0.28, p = 0.11). There were no other significant correlations (for example, with the premotor cortex). Furthermore, there were no significant correlations of reaction times with regions of interest.

### FMRI results: Brain responses while evaluating products after lying in a written note

We then examined brain responses after lying in a written message while assessing products. Lying relative to writing the truth revealed activation in bilateral somatosensory cortex, right premotor cortex (BA6) and BA44 (masked, unmasked analysis revealed no additional results) when participants assessed hand soap products. As a further control condition we calculated brain responses for the same contrast, but masked with the contrast *mouth-rest*. As expected, results revealed no significant voxels. When assessing toothpaste and mouthwash products (after lying in a written message), results revealed no significant results (masked and unmasked) (see Table 3 and Fig. 3).

The opposite contrasts (ethical relative to unethical) failed to reveal significant activations (masked und unmasked) both for assessing mouthwash and hand soap products (after lying in a written note).

Hence, the results showed that immoral acts performed with the hand (writing a note) resulted in sensorimotor activation only when participants rated hand wash products, not when being asked to judge toothpaste and mouthwash products. This sensorimotor activation was in a similar brain region compared with the brain activity while performing the writing (the hand area of the sensorimotor topography, see Fig. 4).

We then compared the effect of lying in a written message on the factor set of products. Results revealed stronger activation of sensorimotor cortex (including SI, M1, premotor cortex), hippocampus, and occipital cortex when participants judged hand wash products compared with toothpaste and mouthwash products. The reverse contrast revealed no significant activations (see Table 2 and Figs 4 and 5). Thus, the Macbeth effect after writing a malevolent message worked for hand wash products, but not for toothpaste and mouthwash products. However, we did not find a significant correlation between signal changes in sensorimotor peak areas (SI and M1, associated with ratings for hand wash products relative to toothpaste and mouthwash products after lying in a written mail) with behavioral responses (p > 0.10, Pearson, two-sided). This can be explained by the only small differences for this contrast in the behavioral data, which failed to reach the level of significance (see above in behavioral results).

There were no other significant correlations. Furthermore, there were no significant correlations of reaction times with regions of interest.

## Discussion

The theory of embodied cognition makes clear assumptions of an interaction between body and mind. The current study aimed to test these assumptions by examining neural mechanisms that underlie embodied knowledge. In particular, we aimed to determine the role of the sensorimotor cortices in the moral-purity metaphor. In line with previous studies we report that people find purification of the “dirty” body part more desirable than purification of other body parts, thus demonstrating that moral purity is specific to the sensorimotor modality involved in moral transgression. FMRI results provide support for the assumptions of the theory of embodied cognition by showing that this interaction was associated with activation especially in the somatosensory cortices (SI). This engagement of SI was somatotopically organized, thus activating mouth areas while evaluating products after lying in a voicemail (but only for toothpaste and mouthwash products) and hand areas after lying in a written note (but only for hand wash products). In addition, the hand area in SI was not recruited for the verbal condition and the mouth area not for the hand condition, thus demonstrating a somatotopic specifity and a double dissociation of the effects.

The theory of embodiment argues that cognition is deeply rooted in sensory experiences. According to this theory social cognitions (e.g., the moral appropriateness of actions) are strongly based on bodily sensations^17^. Numerous behavioral studies confirm the theory of embodied cognitions (e.g.,^7^,^18^). Studies using neuroimaging approaches offer the chance to provide additional support by showing potential engagement of sensorimotor brain areas involved in embodied cognitions, which could proof the assumptions of the theory in a direct way.

Several studies employing fMRI report an involvement of somatosensory brain areas, in particular SI, when examining neural correlates of embodied metaphors. The current study is the first one demonstrating that metaphors are topographically reflected in the somatosensory homunculus, thus providing strong support for the theory of embodiment in general as well as for a fundamental role of SI in this theory in particular.

Our behavior results confirmed the study by Lee and Schwarz^2^ in a with-in subjects design. First, we replicated the Macbeth effect for both modalities. Thus, evaluations of purification goods were higher after lying relative to telling the truth, for both modalities. These results are in line with the ones reported by Zhong and Liljenquist^1^ (see also^14^). Second, participants preferred purification of the “dirty” body part over purification of other body parts, thus confirming the results of Lee and Schwarz^2^. Hence, toothpaste and mouthwash products were evaluated more positively after lying in a voicemail but not after lying in a written note, demonstrating that embodiment is sensitive to motor modality. However, hand wash products seem to wash away the sins both for lying in a voicemail as well as for lying in a written message. This result differs from Lee and Schwarz^2^. Nevertheless, Lee and Schwarz^2^ used slightly different products (mouthwash and hand sanitizer). In addition, they used a between-subjects-design with a higher number of participants. Last, participants of our study had another cultural background. One or a combination of these points might explain the lack of specifity in the hand modality in our study.

Analysis of our fMRI data confirmed our behavioral results. The Macbeth effect for the hand modality (lying vs. telling the truth in a written note) was accompanied with activation in sensorimotor brain areas including SI, BA6 and BA44. These brain regions were engaged after immoral relative to moral acts, but only when assessing hand wash products, not when evaluating toothpaste and mouthwash products (thus extending the behavioral results, which failed to find a significant difference here, see previous paragraph). Similarly, the Macbeth effect for the mouth modality (lying vs. telling the truth in a voicemail) was associated with activity in SI and premotor cortex. Again, these brain regions were engaged after immoral relative to moral deeds, but here only when assessing toothpaste and mouthwash products, not when judging the desirability of hand wash products. Thus, neural correlates of the embodied moral-purity metaphor rely on sensorimotor brain regions, in particular SI. This is in line with previous studies investigating other embodied metaphors (e.g.,^11^,^13^,^14^).

Moreover, SI was differentially involved depending on the modality of lying. Thus, lying in a written note resulted in more dorsal activation of the somatosensory homunculus when assessing the hand wash products. This more dorsal activation was in line with the activation of SI during the writing task (the hand area of the homunculus). In contrast, lying in a voicemail resulted in a more inferior activation of the somatosensory map, similar to the activation of SI during the lying (speaking) task (the mouth area of the homunculus). We conclude that the results confirm the hypothesis that embodiment is sensitive to modality and that the neural underpinnings of this sensitivity are reflected in the somatotopical map in SI.

While previous studies already documented a role for somatosensory brain regions, in particular SI, for embodied metaphors (e.g.,^9^,^11^,^12^,^13^,^14^), the present study demonstrates for the first time a functional topographical dissociation of activity in SI depending on the modality used in moral transgression. Hence, SI seems to maintain a central role for embodied metaphors. This role for SI in embodied metaphors is in line with other studies showing an involvement of somatosensory brain areas in social cognitions. Whereas classic studies understood this body map representation in SI as fix and reflecting the physical location of peripheral stimulation in the form of the famous somatosensory homunculus, recent studies challenge this view and suggest a more complex role for SI. For example, numerous studies suggest that the somatosensory cortices are engaged in social perceptions, in particular to empathy (e.g.,^19^,^20^). Furthermore, mirror-like activations in somatosensory cortices when observing others being touched have been reported^21^. These mirror-like activations in the somatosensory cortices have been shown to be linked with the empathic abilities of the observer (e.g.,^22^,^23^,^24^,^25^,^26^). The present study extends these results of a role of SI in social perception by showing that embodied metaphors such as moral purity are based on somatosensory activations in a functional topographic way.

But why are somatosensory brain areas important for embodied metaphors such as moral purity? It has been hypothesized that early experiences with the physical world structure our later understanding or representation of more abstract concepts^7^,^13^,^27^,^28^,^29^. For example, Williams *et al.*^28^ claims that the primary foundation of knowledge is bodily sensation. Thereby, the concept formation process or “scaffolding” must be first based on bodily sensations. Wilson^30^ added an evolutionary argument by arguing that we are “evolved from creatures whose neural resources were devoted primarily to perceptual and motor processing”.

In his neural reuse theory Anderson argues that the cognitive roles played by each region of the brain are various^31^. Brain areas are involved in different neural partnerships depending on tasks and circumstances. According to Anderson neural reuse means a form of neuroplasticity in which neural elements originally developed for one purpose are put to multiple uses^31^. His neural reuse theory points to our brains early-evolving capacity for recurring interaction with our environment. Anderson claims that the abstract use of physical concepts (e.g., using purity as a metaphor for moral behavior) may be an example of how the brain uses old strategies in new ways. In this way, our novel and abstract higher-order cognitive processes may be just recombinations of more simple and basic brain processes.

Beyond activation of sensorimotor activations we also found engagement of hippocampus and amygdala accompanying the Macbeth effect. These structures are well known to be related to memory functions. More in detail, it has been suggested that those memory structures in the medial temporal lobe may bind distributed activated sites in the neocortex that represent a whole memory^32^. A role for memory structures during the Macbeth effect is in line with the assumptions of the embodiment theory.

While we found bilateral engagement of the somatosensory cortices for the hand (soap) condition, the verbal condition seemed to involve in particular the somatosensory cortex of the left hemisphere. This may be explained simply by the fact that we wash both of our hands with soap, but in general brush our teethes only with the dominant (here the right) hand. However, recent studies also draw the attention to a functional asymmetry in the somatosensory cortices especially for social tasks^33^. Therefore, future research is needed to understand the role of hemispheric asymmetry in the neural underpinnings of the Macbeth effect.”

Thus, together with the studies by Zhong and Lillenquist^1^ and Schnall *et al.*^3^, the results of our study demonstrate that moral judgment can be driven by intuitive and contextual processes rather than reasoning and conscious thought^3^. Our results show that the neural underpinnings of these intuitive processes are in particular the somatosensory cortex, an area that is currently also prominently discussed for social perceptions in general (e.g.,^21^). We conclude that the role of this brain region seems to be more complex than previously thought, possibly playing important roles in knowledge acquisition and understanding of others and thereby providing support for the neural reuse theory by Anderson^31^. Hence, our results support the view that the body influences the mind just as the mind influences the body.

## Additional Information

**How to cite this article**: Schaefer, M. *et al.* Dirty deeds and dirty bodies: Embodiment of the Macbeth effect is mapped topographically onto the somatosensory cortex. *Sci. Rep.* **5**, 18051; doi: 10.1038/srep18051 (2015).

## Figures and Tables

**Table 1:** Results of random effects analysis for brain responses when rating different set of products.

	contrast	brain region	peak MNI location(x, y, z)	peakz-value	numberof voxels
**Evaluation of tootbrush and mouthwash products**	after lying > after telling the truth in a voicemail	L SI/premotor cortex(BA6)(L sup. temporal gyrus)(R sup. temporal gyrus)(L hippocampus/amygdala)occipital cortex/cerebellum	−36 −18 32−46 −26 −262 −18 −2−14 −20 −128 −88 4	3.753.353.303.384.87	6761056614
	after telling the truth > after lying in a voicemail	—	—	—	—
**Evaluation of handwash products**	after lying > after telling the truth in a voicemail	—	—	—	—
	after telling the truth > after lying in a voicemail	—	—	—	—

Displayed are activations surviving cluster level correction (p < 0.05, FWE corrected, threshold of p < 0.005 used to define the clusters, masked with speaking task results, L = left hemisphere, R = right hemisphere; in brackets: uncorrected results).

**Table 2:** Results of random effects analysis for brain responses when rating different set of products after lying in a written note and after lying in a voice mail, respectively.

	contrasts	brain region	peak MNI location(x, y, z)	peakz-value	numberof voxels
**After lying in a written note**	soap > toothpaste and mouthwash	L SIL premotor cortex (BA6)R SMA (BA6)L M1L IPCR SIR IPCoccipital cortexL hippocampus/amygdalaR hippocampus/amygdala	−36 −38 48−28 −16 728 8 52−36 −28 58−24 −66 4236 −42 4826 −62 3616 −90 −6−20 −26 −624 −26 −6	4.655.184.954.965.273.664.407.375.304.84	462571050329
	toothpaste and mouthwash > soap	anterior cingulate cortex	4 34 0	3.81	859
**After lying in a voice mail**	soap > toothpaste and mouthwash	—	—	—	—
	toothpaste and mouthwash > soap	L SI L premotor cortex(BA6) L M1 occipitalgyrus (L superior temporalgyrus) (R superiortemporal gyrus)	−44 −16 28−52 −10 54−46 −14 4610 −86 4−54 −14 −458 −16 −4	3.053.973.935.974.204.23	6903911280398

Displayed are activations surviving cluster level correction (p < 0.05, FWE corrected, threshold of p < 0.005 used to define the clusters, not masked, L = left hemisphere, R = right hemisphere; in brackets: uncorrected results).

**Table 3:** Results of random effects analysis for brain responses when rating different set of products.

	contrasts	brain region	peak MNI location(x, y, z)	peakz-value	numberof voxels
**Evaluation of tootbrush and mouthwash products**	after lying > after telling the truth in a written note	—	—	—	—
	after telling the truth > after lying in a written note	—	—	—	—
**Evaluation of handwash products**	after lying > after telling the truth in a written note	R SIR SI/inf. parietal cortex(L SI)(premotor cortex (BA6))(R inferior frontal gyrus)	46 −28 6664 −26 44−34 −40 4624 −14 5054 12 12	3.243.633.083.303.18	824145663112
	after telling the truth > after lying in a written note	—	—	—	—

Displayed are activations surviving cluster level correction (p < 0.05, FWE corrected, threshold of p < 0.005 used to define the clusters, masked with writing task results, L = left hemisphere, R = right hemisphere; in brackets: uncorrected results).
